# Editorial: Role of Mitochondrial Quality Control in Myocardial and Microvascular Physiology and Pathophysiology

**DOI:** 10.3389/fphys.2021.745033

**Published:** 2021-09-08

**Authors:** Amanda Lochner, Hsueh-Hsiao Wang, Russel J. Reiter, Rui Guo, Hao Zhou

**Affiliations:** ^1^Department of Biomedical Sciences, Faculty of Health Sciences, University of Stellenbosch, Stellenbosch, South Africa; ^2^Department of Medicine, Mackay Medical College, Taipei, Taiwan; ^3^The University of Texas Health Science Center at San Antonio, San Antonio, TX, United States; ^4^College of Life Sciences, Hebei University, Baoding, China; ^5^Department of Cardiology, Chinese People's Liberation Army General Hospital, Beijing, China

**Keywords:** mitochondrial fission, mitochondrial fusion, mitochondrial biogenesis, mitophagy, mitochondria-dependent cell death, endothelial cells, cardiomyocytes

Mitochondrial quality control (MQC) involves a series of adaptive responses of mitochondrial morphological alterations and functional modifications, such as mitochondrial fusion, mitochondrial fission, mitophagy, mitochondrial biogenesis, mitochondrial bioenergetics, and mitochondria-mediated death pathways (Akbari et al., 2019; Del Campo, 2019; Shanmughapriya et al., 2020; Wang et al., 2020c). Mitochondrial damage or impaired MQC has been reported to play an important role in regulating the physiology and/or pathology of myocardium and vessels (Heusch, 2019; Hughes et al., 2020; Wang and Zhou, 2020; Wang et al., 2020b). The objective role of the Research Topic “*Role of Mitochondrial Quality Control in Myocardial and Microvascular Physiology and Pathophysiology*” (https://www.frontiersin.org/research-topics/13532/role-of-mitochondrial-quality-control-in-myocardial-and-microvascular-physiology-and-pathophysiology#research-topic-overview) was to gather original research articles and/or reviews to highlight the recent findings regarding the impact of MQC on various cardiovascular disorders. The article “Physical exercise: a novel tool to protect mitochondrial health” by Sorriento et al. reviews the effects of physical activity on cardiac mitochondrial function underlying the ability to modulate specific steps in mitochondrial quality control in both physiological and pathophysiological conditions. Topics were discussed ranged from the effects of exercise on mitochondrial phenotypes, biogenesis, turnover, morphology and respiration to cardiac pathophysiological conditions such as, aging, ischemia/reperfusion injury (I/R), diabetic cardiomyopathy, and anthracyclines dependent heart failure. From these studies, physical exercise emerges as a non-pharmacological tool (“mitochondrial medicine for muscle”) to improve cardiovascular fitness in healthy people as well as to attenuate mitochondrial dysfunction in patients with pathophysiological conditions, particularly cardiac I/R damage.

Although several critical molecules of mitochondrial quality control have been identified to improve their function, a drug that specifically targets mitochondria has yet to be developed (Jusic and Devaux, 2020; Larson-Casey et al., 2020; Martínez-Milla et al., 2020; Wang et al., 2020a). A number of promising mitochondria-targeted agents have been studied during myocardial I/R, but none of these exhibited sufficient efficacy for clinical use (Hohendanner and Bode, 2020; Ni et al., 2020; Paik et al., 2020; Zhou et al., 2020). The role of the SERCA2a/Ca^2+^-Mfn2 pathway in myocardial ischemia was investigated by Tian and Zhang. By isolation of cardiac microvascular endothelial cells (CMECs) from heart tissues, they found that hypoxia induced irreversible oxidative modifications of SERCA2a, cytosolic and mitochondrial Ca^2+^ overload, mPTP opening and membrane potential disruption were attenuated by either SERCA2a overexpression or Mfn2 ablation. Mfn2 knockout also suppressed mitochondrial fission and Parkin/PINK dependent mitophagy. Thus, their study showed that ablation of Mfn2 rendered the heart resistant to ischemic injury, reduced cardiac microcirculatory damage, suggesting that Mfn2 inhibition during acute myocardial ischemia injury could be a novel cardioprotective strategy. In contrast to this finding, Liu et al. further observed that Mfn2 overexpression was able to attenuate cardio-cerebrovascular ischemia/reperfusion injury through activation of mitochondrial fusion in a manner dependent on the AMPK/Sirt3 pathway. The beneficial actions of Mfn2-controlled MQC were also confirmed by Xiao et al. in a model of hyperglycemia in cardiomyocytes.

In addition to mitochondrial fission or fusion, the role of mitophagy was also discussed by Li et al. in a model of high-fat-induced endothelial dysfunction. They reported that activation of Bnip3-related mitophagy was associated with decreased mitochondrial oxidative stress and increased mitochondrial bioenergy production. Similar to these findings, Xin et al. reported that hypoxia-mediated cardiomyocyte damage could be attenuated by Opa1-related mitophagy through improving MQC. Lastly, in a review summarized by Chang et al. discussed the potential natural drugs targeting MQC in the treatment of cardiovascular disorders. Natural medicines or Chinese herbal medicines have special advantages in the treatment of cardiovascular diseases through multiple and complex mechanisms. This review expands our perspectives and promotes the development of new tools or compounds for future preventive and therapeutic strategies in order to reduce the adverse cardiovascular events. Besides, Chang et al. depicts a promising field that places the interaction between MQC and natural drugs at the forefront of the cardioprotection field to extend lifespan.

In summary, these articles and reviews presented in the Research Topic lay a foundation for us to better understand the role of MQC in myocardial and microvascular pathophysiological conditions. This may highlight a new entry point for treating cardiovascular diseases by targeting MQC.

## Author Contributions

AL, H-HW, and RR: conceptualization. AL, RG, and HZ: writing and original draft preparation and writing review and editing. All authors contributed to the article and approved the submitted version.

## Funding

This work was supported in part by the NSFC (No. 81900252).

## Conflict of Interest

The authors declare that the research was conducted in the absence of any commercial or financial relationships that could be construed as a potential conflict of interest.

## Publisher's Note

All claims expressed in this article are solely those of the authors and do not necessarily represent those of their affiliated organizations, or those of the publisher, the editors and the reviewers. Any product that may be evaluated in this article, or claim that may be made by its manufacturer, is not guaranteed or endorsed by the publisher.
